# Surgical approaches and outcomes for cervical myelopathy with increased signal intensity on T2-weighted MRI: a meta-analysis

**DOI:** 10.1186/s13018-019-1265-z

**Published:** 2019-07-18

**Authors:** Yuan Xu, Feng Chen, Yipeng Wang, Jianguo Zhang, Jianhua Hu

**Affiliations:** 1 Department of Surgery, Peking Union Medical College Hospital, Chinese Academy of Medical Science & Peking Union Medical College, Beijing, China; 2 Department of Orthopedics, Peking Union Medical College Hospital, Chinese Academy of Medical Science & Peking Union Medical College, Beijing, China

## Abstract

**Objective:**

Increased signal intensity (ISI) on T2-weighted magnetic resonance imaging (MRI) often indicates severe compression in patients with cervical myelopathy (CM). The optimal surgical approach for CM patients with ISI on T2-weighted MRI remains unclear. This meta-analysis aims to compare the clinical outcomes between anterior and posterior approaches for the treatment of these patients.

**Methods:**

MEDLINE, EMBASE, Web of Science, and Chinese National Knowledge Infrastructure (CNKI) were searched for relevant studies through January 2019. Statistical comparisons were made when appropriate.

**Results:**

A total of 9 studies (748 participants) out of 1066 citations were included in this study. All of the selected studies were high quality, as indicated by the Newcastle–Ottawa scale and the Cochrane Collaboration tool for assessing the risk of bias. Clinical outcomes were compared between anterior and posterior approaches in 4 studies (237 participants). The preoperative Japanese Orthopedic Association (JOA) score was similar between the two groups [*P* = 0.98, weighted mean difference (WMD) = 0.01 (− 0.58, 0.59)]. The postoperative JOA score [*P* < 0.05, WMD = 0.68 (0.06, 1.30)] and recovery rates [*P* < 0.01, WMD = 0.12 (0.06, 0.17)] were significantly higher in the anterior group than in the posterior group.

**Conclusion:**

The anterior approach was associated with better postoperative neural function than the posterior approach in CM patients with ISI on T2-weighted MRI.

## Introduction

Cervical myelopathy (CM) is a common cause of progressive spinal cord dysfunction. The most common etiology for CM is spinal stenosis caused by cervical spondylosis and ossification of the posterior longitudinal ligament (OPLL) [1]. Surgical treatment aims to expand the cervical canal and relieve the compression of the spinal cord. There are two main surgical approaches: the anterior approach and the posterior approach. Anterior approaches, which can achieve direct decompression by directly removing the ventral stenosing focus, typically comprise anterior cervical discectomy and fusion (ACDF) and anterior cervical corpectomy decompression and fusion (ACCF). Posterior approaches, which can achieve indirect decompression by shifting the spinal cord posteriorly, typically comprise laminoplasty or laminectomy. The choice of the surgical approach for CM has been a controversial issue. Several recent meta-analyses concluded that the anterior approach is associated with better postoperative neurological outcomes than the posterior approach [2–4].

Magnetic resonance imaging (MRI) of the cervical spine is widely used in the diagnosis and preoperative evaluation of CM [5]. Although increased signal intensity (ISI) on T2-weighted MRI often indicates more severe compression, its prognostic value has been debated in several articles with conflicting results, and the optimal surgical approach for these patients remains unclear. The purpose of this study was to perform a meta-analysis of surgical approaches for the treatment of patients with CM with ISI on T2-weighted MRI and to specifically evaluate the clinical results to guide clinical decision-making.

## Methods

### Inclusion and exclusion criteria

Studies were included if they met the following inclusion criteria: (1) the study was a randomized or nonrandomized controlled study (for prospective studies) or a retrospective study, (2) patients were diagnosed with CM due to cervical spondylotic myelopathy (CSM) or OPLL, (3) patients underwent surgical treatment, (4) comparative data between anterior and posterior approaches (regardless of the specific surgical approach) were available, (5) T2 ISI information was available for all or subgroups of patients, and (6) the study had an average follow-up time of ≥ 12 months. Studies were excluded if (1) patients were diagnosed with CM resulting from another etiology, such as trauma, a tumor, or rheumatoid arthritis; (2) patients underwent related thoracic or lumbar surgeries; or (3) the full text could not be accessed.

### Search strategy

MEDLINE, EMBASE, Web of Science, and Chinese National Knowledge Infrastructure (CNKI) were searched through January 2019. The search was not restricted to any specific language or by the year of publication. The following search terms and strategies were used: (1) CM OR CSM OR myelopathy OR cervical spondylosis OR cervical vertebrae OR cervical stenosis OR OPLL OR ossification of posterior longitudinal ligament, (2) corpectomy OR ACDF OR ACCF OR anterior cervical corpectomy decompression and fusion OR anterior cervical discectomy and fusion OR anterior decompression OR ventral decompression OR ventral approach, (3) laminoplasty OR laminectomy OR posterior decompression OR posterior decompression and fusion OR dorsal decompression OR dorsal approach, (4) MRI OR T2 OR T2WI OR signal intensity OR magnetic resonance imaging, and (1) and (2) and (3) and (4). Data were extracted with a standardized form [6]. The Newcastle–Ottawa Scale (NOS) was used for quality assessment of nonrandomized studies [7], and the Cochrane Collaboration tool for assessing risk of bias [8] was used for randomized studies. Two reviewers independently reviewed the articles in the initial and full-text reviews. Any disagreement between reviewers was resolved by discussion with a third reviewer.

### Statistical analysis

Inconsistency between studies was quantified by calculating the *I*^2^ statistic. Continuous variables are reported as weighted mean differences (WMDs) and 95% confidence intervals (95% CIs), whereas dichotomous variables are presented as odds ratios (ORs) and 95% CIs. A random-effects model was used for heterogeneous data (*I*^2^ > 50%), whereas a fixed-effects model was used for homogenous data (*I*^2^ < 50%). For studies presenting data as medians and quartiles, the mean and standard deviation (SD) were calculated by methods described by Hozo et al. [9]. *P* < 0.05 was considered to be statistically significant. SAS software, version 9.1 (SAS Institute, Cary, NC, USA), and Review Manager, version 5.1 (The Cochrane Collaboration), were used to perform statistical analysis.

## Results

### Search results

**Fig. 1:**
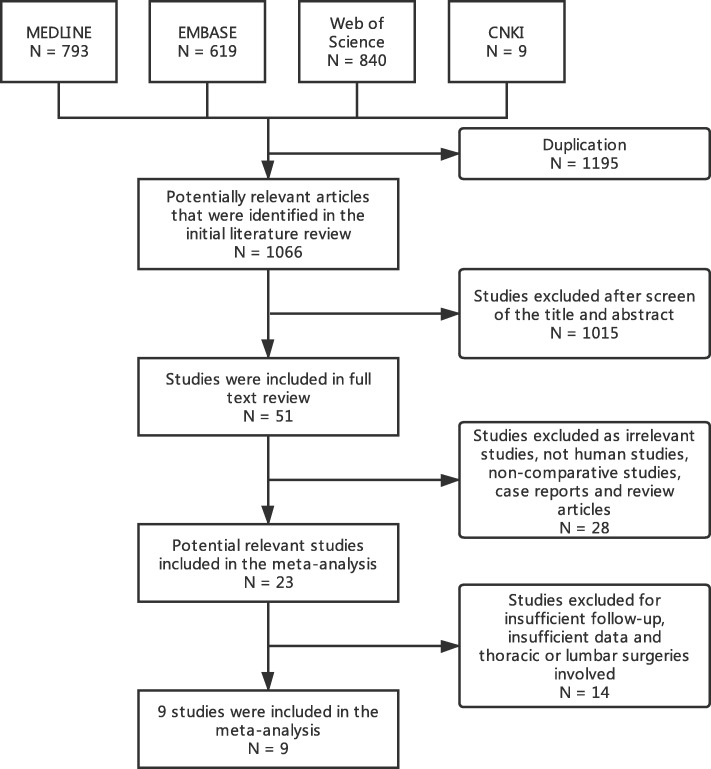
A flow diagram of study selection

The initial database search identified 793 articles in MEDLINE, 619 in EMBASE, 840 in Web of Science, and 9 in CNKI. After excluding duplicate records, there were 1066 studies. A total of 1015 articles were excluded because they failed to meet the inclusion criteria after review of the abstracts and titles. An additional 42 articles were excluded after the full-text review. Hence, a total of 9 studies were finally selected, including 1 prospective randomized study, 6 prospective nonrandomized studies, and 2 retrospective studies. The detailed selection process is shown in Fig. 1.

### Quality assessment

The basic characteristics of the included studies are shown in Table 1. The quality of the included nonrandomized studies was assessed using the NOS (http://www.ohri.ca/programs/clinical_epidemiology/oxford.htm). Two independent reviewers conducted the assessment. Disagreements were resolved by discussion. Of the nonrandomized studies, four scored 8 points, two scored 7 points, and two scored 6 points (Table 2). The Cochrane Collaboration tool for assessing risk of bias was used to assess the randomized study (one study), as shown in Figure S1. The assessments indicated that all of the studies were of relatively high quality.

**Table 1:** Basic characteristics of the included studies

ID	Location	Design	Enrollment years	No. with T2 ISI	Classification of T2 ISI	Follow-up time (month)	Diagnosis	Segments	Surgical approach	No. using approach
Gu et al. [10]	China	Prospective nonrandomized controlled	2010–2012	140	NM	27 (12–360)	OPLL	2–6	A: ACCF	27
									P: Laminectomy, Laminoplasty	113
Sun et al. [11]	China	Prospective randomized controlled	2005–2008	31	NM	20.3 (12–34)	OPLL	NM	A (NM)	15
									P: Laminoplasty	16
Liu et al. [12]	China	Prospective nonrandomized controlled	2002–2006	17	NM	81.6 (60–120)	OPLL	NM	A: ACCF	7
									P: Laminoplasty	10
Wang [13]	China	Prospective nonrandomized controlled	2000–2007	49	Qualitative	13.8 (12–60)	CSM	NM	A:ACCF	29
									P: Laminoplasty	20
Yang et al. [14]	China	Prospective nonrandomized controlled	2010–2014	202	NM	36 (28–40)	OPLL	≥ 2	A:ACCF, ACDF, HDF	121
									P: Laminoplasty	81
Salem et al. [15]	The UK	Prospective nonrandomized controlled	2006–2010	66	NM	37 (17–88)	CSM	NM	A:ACCF, ACDF, ACDR	24
									P: Laminoplasty	42
Suri et al. [16]	India	Prospective nonrandomized controlled	1999–2001	121	NM	12–24	CSM	≥ 1	A: ACCF, ACDF	NM
									P: Laminectomy, Laminoplasty	
Tauchi et al. [17]	Japan	Retrospective	1991–2010	21	NM	32.4 (12–153)	CSM	NM	A:ACCF, ACDF, HDF	NM
									P: Laminectomy, Laminoplasty	
Uchida et al. [18]	Japan	Retrospective	1988–2001	101	Qualitative	99.6 (12–154)	CSM, OPLL	≥ 1	A: ACCF, ACDF	NM
									P: Laminoplasty	

*NM*, not mentioned

**Table 2:** Quality assessment according to the Newcastle–Ottawa scale

ID	Selection	Comparability	Exposure	Total score
Gu et al. [10]	3	2	3	8
Liu et al. [12]	3	2	3	8
Wang [13]	2	2	3	7
Yang et al. [14]	3	1	3	7
Salem et al. [15]	2	1	3	6
Suri et al. [16]	3	2	3	8
Tauchi et al. [17]	2	1	3	6
Uchida et al. [18]	3	2	3	8

### Surgical approaches

The 9 studies included a total of 748 patients with ISI on T2-weighted MRI. Data on the surgical approach were extracted for 505 patients, among whom 223 underwent an anterior approach, including ACCF, ACDF, anterior cervical disc replacement (ACDR), or hybrid anterior decompression and fusion (HDF), and 282 underwent a posterior approach, including laminoplasty or laminectomy.

### Clinical outcomes

**Fig. 2:**
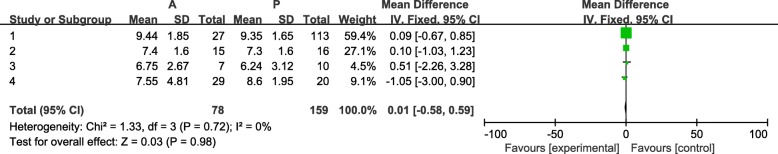
Weighted mean difference in the preoperative JOA score between the anterior surgery group and the posterior surgery group

**Fig. 3:**
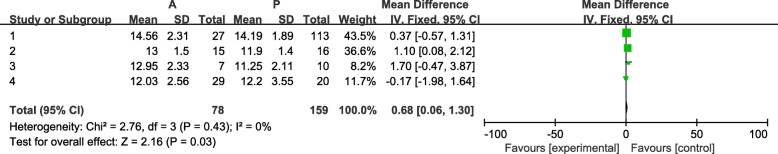
Weighted mean difference in the postoperative JOA score between the anterior surgery group and the posterior surgery group

**Fig. 4:**
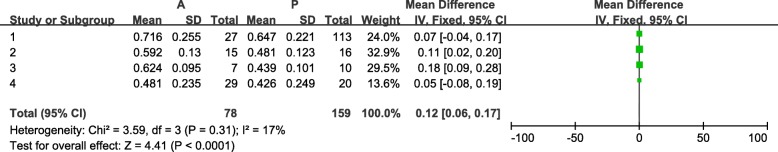
Weighted mean difference in the recovery rate between the anterior surgery group and the posterior surgery group

The Japanese Orthopedic Association (JOA) score was available in 4 studies to assess the clinical outcome (*n* = 237 patients; 78 in the anterior group and 159 in the posterior group). The preoperative JOA score was similar between the two groups [*P* = 0.98, WMD = 0.01 (− 0.58, 0.59); heterogeneity: *χ*^2^ = 1.33, df = 3, *P* = 0.72; *I*^2^ = 0%, fixed-effects model; Fig. 2]. The postoperative JOA score was significantly higher in the anterior group than in the posterior group [*P* < 0.05, WMD = 0.68 (0.06, 1.30); heterogeneity: *χ*^2^ = 2.76, df = 3, *P* = 0.43; *I*^2^ = 0%, fixed-effects model; Fig. 3]. The recovery rate, as defined by Hirabayashi’s formula: (postoperative JOA score − preoperative JOA score)/(17 − preoperative JOA score) × 100%, was used to assess the surgical outcome. Similar to the results of the postoperative JOA score, the recovery rate was significantly higher in the anterior group than in the posterior group [*P* < 0.01, WMD = 0.12 (0.06, 0.17); heterogeneity: *χ*^2^ = 3.59, df = 3, *P* = 0.31; *I*^2^ = 17%, fixed-effects model; Fig. 4]. Data regarding complications were scarce. Only Sun’s study reported that the anterior approach was associated with more blood loss and a longer operative time [11].

## Discussion

In the present study, we searched MEDLINE, EMBASE, Web of Science, and CNKI. We compared surgical outcomes of anterior and posterior surgical approaches in CM patients with ISI on T2-weighted MRI. We showed that the anterior approach was associated with better recovery than the posterior approach, as assessed by the JOA score.

CM is a common cause of spinal cord dysfunction in older persons. Since Takahashi first reported ISI on T2-weighted MRI in a patient with CM [19], MRI has been recognized as essential for the diagnosis and treatment of CM. It is documented that 41–97% of patients with CM have ISI on T2-weighted MRI within the cervical cord [20]. The role of signal intensity changes on MRI has been widely investigated. There are several theories explaining ISI on T2-weighted MRI: (1) myelomalacia caused by spinal edema or chronic spinal cord compression, (2) destruction of the local cerebrospinal fluid (CSF) barrier, (3) obstruction of the flow of CSF, and (4) degeneration of gray matter at the level of compression level [21, 22]. Several studies have suggested that cervical instability is a risk factor for ISI on T2-weighted MRI because dynamic factors may result in spinal cord injury [23–25]. Moreover, Yagi et al. suggested that males were more likely to have ISI on T2-weighted MRI than females (OR = 3.348), although the difference was not significant (*P* = 0.101) [26]. This finding may result from the higher morbidity of developmental cervical spinal canal stenosis in males than in females.

Several studies have suggested that patients with ISI have poor clinical results after either conservative or surgical treatment. However, some other studies have challenged this hypothesis [27]. To clarify this question, several studies have classified signal intensity changes via different methods and studied their relevance to clinical outcomes. Aditya et al. categorized the various classification methods into two major types: the longitudinal extent of ISI and qualitative classification of ISI [20]. Studies using the longitudinal extent to classify ISI have usually suggested that patients with multisegmental ISI have more severely compressed cords [28], poorer surgical outcomes [29], and worse recovery rates [30] than those with focal or absent ISI. The qualitative classification of ISI involves an assessment of the intensity, marginal pattern, or both. Studies using qualitative classification are controversial. Most studies have indicated that sharp, more intense ISI is correlated with worse clinical outcomes [31–35], whereas other studies have suggested that the intensity of ISI on T2-weighted images does not predict surgical outcomes [27, 36].

As there are usually multiple sources of compression from the anterior and/or posterior sides, several factors should be taken into consideration when choosing the approach. MRI findings play an important role in decision-making. Nouri et al. conducted a questionnaire survey among 513 AOSpine International members to understand how specific pathologic features on MRI influence the surgeon’s selection of a surgical approach selection in patients with degenerative CM [37]. Multilevel bulging disks, cervical kyphosis, and a high degree of anterior cord compression have been reported to lead to selection of an anterior approach, whereas a high degree of posterior cord compression, multilevel compression, OPLL, ligamentum flavum enlargement, and congenital stenosis have been reported to lead to selection of a posterior approach [2, 3, 37]. Although the presence of ISI on T2-weighted MRI is not considered an important factor that influences decision-making, interviewers were slightly more likely to choose the anterior approach than the posterior approach (23.4% vs. 15.6%, respectively).

In the present study, the postoperative JOA scores and recovery rates were significantly higher following the anterior approach than following the posterior approach, suggesting that the neurological outcomes were better with the anterior approach. Although there have been no meta-analyses or systemic review studies focusing on the surgical approach for CM patients with ISI on T2-weighted MRI, there are similar studies regarding the surgical approach for all CM patients [2, 38–40]. In the study by Chen et al., a total of 25 nonrandomized controlled studies involving 1843 patients were included [2]. Similarly, the anterior approach was found to be associated with better postoperative neurological outcomes than the posterior approach in CSM patients. However, several studies, including one meta-analysis [40], have suggested that current studies are unable to demonstrate clinically significant differences between anterior and posterior approaches. Our study showed a 12% higher recovery rate following the anterior approach than following the posterior approach. Since ISI often indicates severe compression, the anterior approach is effective in releasing the compressed spinal cord in patients with ISI. Compared with the posterior approach, the anterior approach can decompress the spinal cord directly and allow substantial restoration of cervical lordosis. This may also explain why studies on surgical approach selection for all CM patients are controversial—in terms of the patients with mild CM, both the anterior and posterior approach can provide sufficient space for a compressed spinal cord.

Surgical complications of CM include dysphagia, airway compromise, dysphonia, C5 nerve palsy, blood loss, and postoperative wound infection. For all CM patients (regardless of T2-weighted imaging findings), no significant differences in complication rates and revision rates have been observed in previous studies when comparing the anterior approach with the posterior approach [41–43]. Data for patients with ISI on T2-weighted MRI are scarce, and the available studies did not report the incidence rate in detail. Sun et al. suggested that OPLL patients with ISI on T2-weighted MRI had more blood loss and a longer operative time than those without ISI [11]. This outcome may be because anterior exposure of the cervical spine involves more complex and potentially dangerous anatomy, especially for patients with ISI on T2-weighted MRI. However, more data are needed to draw a conclusion.

Our study has several limitations. First, the disparity in surgical interventions was not considered—i.e., ACCF vs. ACDF, laminectomy vs. laminoplasty—although postoperative outcomes have been suggested to be similar in previous studies [44, 45]. Second, most of our included studies were nonrandomized. Although the quality of the included studies did not seem to be poor based on an assessment with the NOS or the Cochrane Collaboration tool, more randomized controlled trials are needed due to the variety of procedures available and the spectrum of results. Third, as the CNKI contains only Chinese literature, the use of this database might have resulted in selection bias. Moreover, most studies commented sparsely about specific inclusion and exclusion criteria.

## Conclusion

In conclusion, the anterior approach was associated with better postoperative neural function than the posterior approach in CM patients with ISI on T2-weighted MRI. The complication rate requires further investigation.

## Data Availability

The datasets used and/or analyzed during the current study are available from the corresponding author on reasonable request.

## Abbreviations

ACCF: Anterior cervical corpectomy decompression and fusion
ACDF: Anterior cervical discectomy and fusion
ACDR: Anterior cervical disc replacement
CI: Confidence interval
CM: Cervical myelopathy
CNKI: Chinese National Knowledge Infrastructure
CSF: Cerebrospinal fluid
CSM: Cervical spondylotic myelopathy
HDF: Hybrid anterior decompression and fusion
ISI: Increased signal intensity
JOA: Japanese Orthopedic Association
MRI: Magnetic resonance imaging
OPLL: Ossification of the posterior longitudinal ligament
OR: Odds ratio
SD: Standard deviation
WMD: Weighted mean difference
